# Clinical Significance of MicroRNA Expression Profiles and Polymorphisms in Lung Cancer Development and Management

**DOI:** 10.4061/2011/780652

**Published:** 2011-07-27

**Authors:** Francesca Megiorni, Antonio Pizzuti, Luigi Frati

**Affiliations:** ^1^Department of Experimental Medicine, Sapienza University of Rome, Viale Regina Elena, 324-00161 Rome, Italy; ^2^Department of Molecular Medicine, Sapienza University of Rome, Viale Regina Elena, 324-00161 Rome, Italy

## Abstract

Lung cancers account for a huge percentage of death in industrialized countries, and hence there is an increasing call for the development of novel treatments. These malignancies are caused by a combination of environmental factors, principally cigarette smoking and genetic alterations. MicroRNAs (miRNAs) are a recently discovered class of regulatory noncoding small RNAs with a significance in numerous biological processes. Strong evidence links miRNA impaired expression profiles and pathways to the etiology of several diseases, including neoplasia. This paper focuses on the emerging role of miRNA function in lung cancer development with particular highlighting on the use of miRNA profiles and polymorphisms for the molecular and biological characterization of tumor pulmonary growth and progression. Furthermore, we underline the potential utility of lung cancer-associated miRNAs as clinical biomarkers with a diagnostic, prognostic, and therapeutic significance and give emphasis to the promising novel miRNA-based curative strategies.

## 1. Introduction

Lung cancer is one of the commonest neoplasia and the first cause of death worldwide, in both women and men, with an increasing incidence rate. Less than 10% of people with the disease live longer than five years after diagnosis [1, 2]. Lung tumor is characterized by a preponderance of carcinoma derived from changes and abnormally growth of epithelial lung cells. Small cell lung carcinoma (SCLC) and non-small cell lung carcinoma (NSCLC) are two main distinct types of this neoplasia that have considerable differences in pathogenetic mechanisms, cellular origin, molecular changes and histopathological and clinical features [1]. Furthermore, they show a different response to therapeutic treatments such as surgical resection, radiation, and chemotherapy. NSCLC comprises approximately 80% of all lung cancers and can be classified into adenocarcinoma (AC), squamous cell carcinoma (SqCC), and large cell carcinoma, whereas SCLC tends to spread more quickly than NSCLC and can be divided into small cell carcinoma (SCC), mixed small cell/large cell carcinoma, and combined small cell carcinoma. Symptoms can be absent or very moderate, mainly in the early stages of tumor transformation, so that a large percentage of patients is diagnosed only in advanced stage of tumor extension with a consequent poor treatment outcome. Although surgery provides a potential curative strategy, patients often develop recurrence with a survival rate that remains very low especially in subjects with metastatic disease. Therefore, looking for biomarkers able to accurately detect early tumor changes is the principle warrant in lung cancer management [3]. Different factors have been associated with an increased risk of lung cancer development, mainly cigarette smoke and air-carcinogens, as well as a family history of pulmonary tumor. Lung neoplasia has indeed a multifactorial etiology being caused by gene-gene and gene-environment interactions [4, 5]. The importance of genetic background in lung tumor has been well highlighted and, in recent years, microRNA- (miRNA or miR) specific signatures have been reported to play a critical role in cancer transformation. These molecules represent a novel potential class of sensitive markers for different and significant clinical applications. 

## 2. MicroRNA Overview

MicroRNAs are evolutionarily conserved small noncoding RNAs that negatively regulate gene expression at the posttranscriptional level by repressing translation or decreasing mRNA stability [6, 7]. MiRNAs are initially transcribed by RNA polymerase II as long primary molecules (pri-miRNAs) that are processed in the nucleus into hairpin precursors (pre-miRNAs) via the RNase-III enzyme DROSHA and the RNA-binding protein DGCR8 [8]. Pre-miRNAs are further transported to the cytoplasm by RAN GTPase/Exportin 5 complex where they are matured into active miRNAs by RNase III enzyme Dicer [9]. Mature miRNA, a single-stranded RNA with a length of about 22 nucleotides, is incorporated into an RNA-induced silencing complex (RISC) by RNA processing proteins, such as AGO1 and AGO2 [10]; miRNA is now able to bind target transcripts through base pairing with their 3′-untranslated regions (UTRs). The sequence important for mRNA recognition encompasses bases 2 to 8 at the 5′-end of the mature miRNA, and it is known as the “seed sequence” [6]. 

To date, more than 800 miRNAs have been identified in the human genome [11], each of them having the potential capacity to bind to hundreds of transcripts, and the biological functions of most miRNAs are rapidly emerging. As estimated by *in silico* prediction algorithms [11, 12], miRNAs regulate at least 30% of the protein-encoding genes and are involved in a broad range of cellular processes such as proliferation, differentiation, homeostasis, and apoptosis, so it is not strange that the dysregulation into the miRNA pathway might contribute to human diseases, including cancer development. Distinct patterns of miRNA expression share common pathways and display a significant tumor specificity supporting their use as promising clinical and biological markers for different cancer aspects as formation, progression, diagnosis, prognosis, and response to therapy [13–17].

### 2.1. MiR-Profiling in Lung Cancer

Since their discovery, increasing evidence indicates a deep involvement of miRNAs in human lung tumorigenesis by acting as tumor suppressors or oncogenes, largely by targeting proteins that are key regulators of cellular proliferation and survival, DNA repair, and immune response [18]. Expression of specific microRNAs is substantially changed in a number of different lung cancer types compared to nontumor lung tissues and cells [19, 20]. Furthermore, many of these miRNAs are located at chromosome regions which are frequently deleted or amplified in several malignancies [13, 21]. *Let-7* was the first microRNA reported to have a role in lung cancer development [22]. Reduced levels of *let-7* miRNA family members were found both *in vitro* and *in vivo* studies of human pulmonary neoplasia, and *let-7*-mediated tumor suppression was suggested to occur largely through the regulation of *HMGA2* and *RAS* genes associated with cell division [20, 22–24]; *miR-126* expression in different lung tumor cell lines was inverse to the upregulated *VEGF* which resulted to be a direct target of *miR-126* [25]. Chen et al. [26] showed that *miR-145* amount was markedly decreased in NSCLC tumor specimens and in two human NSCLC cell lines, A549 and H23, relative to nonmalignant lung tissues and cells, respectively. *MiR-145* has been shown to act as an antioncogenic molecule able to inhibit proliferation and the G1/S transition of lung cancer cells by affecting c-Myc/eIF4E4/CDK4 protein levels without changes in apoptosis [27]. Dysregulation of *miR-451* correlated with the development of non-small cell lung cancer by enhancing apoptosis probably through the inactivation of Akt signaling pathway [28]. In particular, downregulation of *miR-451* was significantly associated with strong positive immunoreactivity of RAB14 protein, a member of RAS oncogene family. Although the majority of miRNAs have been proposed to operate as tumor suppressors in lung cancer, as suggested by their overall downregulation, an increasing number of miRNAs have been found to have oncogenic properties. Overexpression of *miR-17-92*, *miR-31*, *miR-21*, and *miR-7* significantly enhanced proliferation and tumorigenicity of lung cancer cells largely by inhibiting negative regulators of oncogenic pathways [20, 29–32]. 

Moreover, miRNA expression signatures strictly correlate with tumor development and progression, also at an early stage, and give significant improvement in the molecular characterization and biological classification of a wide variety of human cancers, including lung malignancy [15, 33, 34]. For example, Yanaihara et al. [19] showed that a subset of miRNAs was differently expressed in adenocarcinomas and squamous cell carcinomas, with *miR-99b* and *miR-102* showing higher levels in ACs. Overexpression of *miR-205* was only detected in SqCCs but not in ACs, and an accurate distinction of the two NSCLC types was allowed by monitoring its levels just in the early phases of cancer transformation [35]. It has been speculated that the use of microRNAs as tumor stage markers might also avoid the variability in the pulmonary cancer subclassification observed with the conventional histopathologic and immunohistochemical techniques due to quality/quantity limitations of bioptic material and staining interpretation [15, 33, 36, 37]. The definition of lung carcinoma subclasses with respect to miRNA quantification is extremely sensitive and specific owing the high stability of miRNAs even in poorly conserved specimens and to the advanced technologies used for their detection as Real-Time PCR and Microarray [15]. 

MiRNA profiling was also linked to clinicopathological tumor features and lung cancer patient outcome, thus having a potential diagnostic and prognostic impact [38–40]. Downregulation of *miR-125a-3p* and *miR-125a-5p* in non-small cell lung carcinomas predicted a more aggressive clinical course by promoting tumor invasion and lymph node metastasis [41]. Low levels of *let-7a-2* in combination with specific expression changes in other microRNAs associated with poor survival in patients with lung adenocarcinoma [19, 38]. The expression level of *miR-451* was found to be significantly associated with NSCLC tumor differentiation, pathological stage, lymph node metastasis, and shorter overall survival of patients [28]. Elevated levels of *miR-21*, *miR-17*, and *miR-155* were associated with cancer-specific mortality and disease-free survival in NSCLC patients from Maryland, Norway, and Japan [42]. In particular, increased *miR-21* expression was found in advanced stage tumors in all the three different cohorts supporting its prognostic value for lung adenocarcinoma. A recent work by Arora et al. [43] gave evidence of the importance to test the expression of *miR-328* in NSCLC samples since it correctly classified patients at higher risk for developing brain metastasis. Moreover, several lines of evidence suggest that microRNA deregulation in lung tumors may also predict disease recurrence and/or response to adjuvant anticancer treatments, such as curative surgery, chemotherapy, and radiotherapy [44, 45]. Downregulation of *microRNA-128b*, due to loss of heterozygosity of 3p22 in lung tumor specimens, was associated to increased epidermal growth factor receptor (*EGFR*) expression with a consequent survival benefit in patients treated with gefitinib, a EGFR-tyrosine kinase inhibitor drug commonly used in advanced NSCLC management [13]. High levels of miR-92a-2* correlated with chemoresistance and decreased survival of SCLC cases [46], whereas *let-7* family of miRNAs could augment sensibility to radiation in lung cancer cells [47]. Furthermore, reduced expression of *let-7* in NSCLC tissues was indicative of elevated postoperative risk of death of the surgically treated patients [22]. Taken together, all these observations suggest that the evaluation of expression changes just in a subset of miRNAs might give important information of practical use in clinical trials for the management of lung oncologic patients since efficacy of therapeutic treatments mainly depends on accurate and early tumor definition [3]. Moreover, microRNA profiling results to have a predictive, diagnostic, and prognostic application that can avoid the use of aggressive tools, such as computed tomography, in lung tumor detection and management [48]. The presence of stably expressed and quantifiable miRNA levels in human serum, which are able to discriminate between normal and lung cancer samples, is an exciting discovery that strengthens the use of these small regulatory molecules as novel noninvasive biomarkers in lung cancer [49–51]. In particular, serum miRNA detection might have the advantage to early select patients for appropriate treatment strategies in order to enhance their survival chance. However, results obtained among different studies suggest that circulating miRNA quantification needs accurate standardization before the potential as a screening tool in the management of lung cancer may be realized. In this regard, an important goal was obtained by Yu et al. [52] that developed a highly sensitive and specific panel of miRNAs for the early detection of lung adenocarcinoma in sputum. In particular, *miR-21*, *miR-486*, *miR-375*, and *miR-200b* showed a significantly different expression in lung adenocarcinoma patients versus normal subjects and their combined values were able to distinguish affected individuals from healthy controls with 80.6% sensitivity and 91.7% specificity in independent populations.

### 2.2. MiR-Polymorphisms and Lung Tumor Susceptibility

New findings have shown that alterations of miRNA expression or activity in cancers can be due to sequence variations in miRNA gene regions, in genes involved in miRNA biosynthesis or at binding sites in the 3′-UTR of miRNA target genes, collectively referred to as “miR-polymorphisms” [17, 53–55]. Single-nucleotide polymorphisms (SNPs) might indeed affect miRNA primary transcript formation and expression, miRNA processing and maturation, and/or miRNA-target interactions. The role of polymorphisms in miRNA sequences and in genes involved into miRNA biogenesis is rapidly emerging and several studies have demonstrated their role in a wide spectrum of malignancies, including lung cancer [54–59]. In particular, miRNA genetic variants might have a potential clinical utility being predictors of lung tumor predisposition. The rs11614913T>C SNP in the *miRNA-196a2* sequence increased mature miRNA expression and target binding activity. Moreover, case-control studies in different ethnic populations showed that the CC genotype was significantly associated to an increased risk of lung cancer, particularly SqCC type, and predicted a reduced patient survival [56, 60]. Kim et al. [61] found that *AGO1* rs636832A>G could modulate susceptibility to lung neoplasia since both the heterozygotes and homozygotes for the G allele had a decreased risk of lung cancer compared with subjects carrying the AA genotype. The genetic variation rs712T>G in a let-7 miRNA binding site in the 3′-UTR of *K-RAS* transcript was found to significantly increase the risk of NSCLC, especially by stratifying individuals for smoking history, through the oncogene upregulation [62]. Additional replication studies in other populations and in vitro experiments for the characterization of the biological significance of these particular SNPs are required to validate these findings. Nevertheless, screening for miR-polymorphisms could lead to better define the genetic risk to lung carcinogenesis as well as its recurrence development. For instance, miR-SNP genotypes and/or haplotypes might allow to categorize lung tumor patients into low, medium, and high-risk groups of disease with respect to determinate clinical aspects, such as cancer progression, chance of survival, and response to therapies. Combinations of various unfavorable SNPs could more accurately identify the risk of cancer for each individual. In a recent work, Rotunno et al. [63] showed that the GTAATC haplotype in *RNASEN*, coding for Drosha, connected with lung cancer-specific reduced survival; moreover, a variant allele of rs640831 was significantly associated with *RNASEN* reduced mRNA expression and with changes in the levels of particular microRNAs only in adenocarcinomas but not in SqCC samples. 

Hence, the potential prognostic implications of microRNA-related genetic variations in lung cancer may be used for developing novel personalized therapeutic applications and for checking patient outcome. 

### 2.3. MiR-Based Therapies in Lung Cancer

The accumulated data on miRNA role as oncogenes or tumor suppressors in lung carcinogenesis suggest that miR-based applications in cancer medicine are an attractive and promising area for patient cure. Indeed, if aberrant microRNA levels are found into tumor tissues, restoring specific microRNA expression might have therapeutic implications. Recent research activities have been focused on the discovery of new molecules able to selectively and stably interfere with microRNA regulatory networks and on the evaluation of the efficacy, stability, tolerability, and cost of the miR-based therapeutic agents. In this context, therapies for knocking down specific miRNAs with oncogenic activity are potentially based on antisense miRNA modified-oligonucleotides, also known as antagomirs, miRNA sponges, miRNA masking, and small molecule inhibitors, while miRNA mimics might be useful in the reintroduction of miRNAs with tumor suppressor capability [14, 64, 65]. Experimental and preclinical data demonstrated that the restoration of cancer-related microRNA expression changes to normal levels by miRNA mimics or antagomirs could reverse the cancer phenotype in tumor cells and in animal models of human neoplasia [66, 67]. In particular, mice intranasally inoculated with adenovirus expressing *let-7a* microRNA showed decreased pulmonary tumor formation in comparison to animals treated with a scrambled sequence [68]. Indication that replacement of *let-7* family members may effectively interfere with lung carcinogenesis *in vivo* was also confirmed by a recent study by Trang et al. [69] in which let-7 intranasal injection of mice led to reduced tumor lung size. Liposomal delivery of chemically synthesized *miR-34a* in mouse models of NSCLC was specifically able to restore microRNA expression, to silence down-stream targets, and to inhibit lung tumor growth without inducing an immune response, a pivotal characteristic for a clinical application in tumor patient treatment [70]. Despite these positive results, several limitations need to be overcome before miRNA-based approaches might be successfully used in human lung cancer treatment either alone or in combination with conventional therapies. Continuous studies are strongly required in order to increase miRNAs specific and efficient tissue delivery, to improve microRNA stability, to keep constant their activity, and to prevent off-target effects, immune stimulation, and toxicity. To this regard, nonviral nanoparticle-based gene/RNA-delivery vectors seem to be a promising system for an efficient miRNA cellular uptake [71]. An efficient transport and delivery was obtained by encapsulating microRNAs into high-density lipoproteins or cationic liposomes [72, 73]. Ligands or monoclonal antibodies that recognize tumor-specific receptors could be conjugated to mimics/antagomirs in order to target miRNAs at the disease site selectively [74, 75]. Strategies for preventing nuclease degradation and increasing miRNA half life are essentially linked to chemical modifications of the RNA backbone such as 2′-fluoro/2′-methoxyethyl groups, phosphorothioate bonds, and nucleobases [76, 77]. In addition, an extremely precise definition of the specific miRNA levels and molecular mechanisms in the different lung cancer types and subtypes is necessary to identify novel targets which might be used for the development of new therapeutic strategies. In this context, interesting studies have demonstrated that DNMT3A and DNMT3B, two DNA methyltransferases involved in epigenetic cellular control, were direct targets of *miR-29s* and their over-expression inversely correlated with *miR-29s* levels in NSCLC, suggesting that drugs able to normalize DNA methylation patterns might be used in clinical trials as lung anticancer agents [78, 79]. 

## 3. Conclusions

Lung cancer is one of the main causes of cancer-related deaths in industrialized countries with only 15% of all patients surviving five years or more. Surgical resection of tumor tissues is the only actual chance of life, together with chemotherapy and/or radiotherapy for some individuals. MicroRNAs are a class of endogenous small regulatory RNAs that negatively control gene expression at the posttranscriptional level. Impaired expression of specific miRNAs has been associated with lung cancer pathogenesis in several studies and microRNA profiles might serve as novel biomarkers to predict cancer progression, prognosis, genetic risk, recurrence, and drug sensitivity (Table 1). Ameliorating the acknowledgements of specific miRNA regulators, tumor functions, downstream targets, and signaling pathways in lung cancer is essential for elucidating complex regulatory networks that are critical for the malignancy biology. These findings will have to be focused on the development of novel preventive and curative treatments. MiRNA mimics or antisense oligonucleotides are indeed promising new tools for cancer therapy.

## Figures and Tables

**Table 1:** Lung cancer-related microRNAs.

MicroRNA	Expression	Target/Function	Clinical value
			
*let-7* family	Down	*HMGA2*, *RAS*, *Myc*, cell division	Associated with cancer-specific mortality and disease-free survival; increased sensibility to radiation; elevated postoperative risk of death; rs712GG genotype in *K-RAS* 3′-UTR associated with increased risk of NSCLC
*let-7a-2*	Down	—	Poor survival in AC patients
*miR-17*	Up	cell proliferation	Associated with tumor stage, cancer-specific mortality, and disease-free survival in NSCLC
*miR-102*	Up	—	Higher levels in AC than in SqCC
*miR-125a-3p/5p*	Down	—	Associated with tumor invasion and lymph node metastasis
*miR-126*	Down	*VEGF*	—
*miR-128b*	Down	*EGFR*	Benefit in patients treated with gefitinib
*miR-145*	Down	*c-Myc*,* eIF4E4*,* CDK4 *	—
*miR-155*	Up	—	Associated with tumor stage, cancer-specific mortality, and disease-free survival in NSCLC
*miR-196a2*	Up	—	rs11614913CC genotype associated with increased risk of lung cancer and reduced overall survival
*miR-20b*	Down	—	Associated with advanced stages and lymph node metastasis
*miR-21*	Down	*K-RAS*, cell proliferation	Associated with tumor stage, cancer-specific mortality, and disease-free survival in NSCLC; able to discriminate lung cancer from healthy controls
*miR-29s*	Down	*DNMT3A, DNMT3B*	—
*miR-205*	Up	—	Only detected in SqCC
*miR-31*	Up	*LATS2*, *PPP2R2A*, cell proliferation	—
*miR-328*	Up	cell migration	Associated with higher risk of brain metastasis development
			Associated with overall survival
*miR-451*	Down	*RAB14*, apoptosis	Associated with NSCLC stage, lymph node metastasis, and poor survival
*miR-7*	Up	*Ets2*, cell proliferation	—
miR-92a-2*	Up	—	Chemoresistance and decreased survival of SCLC cases
*miR-99b*	Up	—	Higher levels in AC than in SqCC

NSCLC: non-small cell lung carcinoma; AC: adenocaercinoma; SqCC: squamous cell carcinoma.
